# HIV-1 Disease Progression Is Associated with Bile-Salt Stimulated Lipase (*BSSL*) Gene Polymorphism

**DOI:** 10.1371/journal.pone.0032534

**Published:** 2012-03-06

**Authors:** Martijn J. Stax, Neeltje A. Kootstra, Angélique B. van 't Wout, Michael W. T. Tanck, Margreet Bakker, Georgios Pollakis, William A. Paxton

**Affiliations:** 1 Laboratory of Experimental Virology, Department of Medical Microbiology, Center for Infection and Immunity Amsterdam (CINIMA) at the Academic Medical Center of the University of Amsterdam, Amsterdam, The Netherlands; 2 Department of Experimental Immunology, Sanquin Research, Landsteiner Laboratory, and CINIMA at the Academic Medical Center of the University of Amsterdam, Amsterdam, The Netherlands; 3 Department Clinical Epidemiology, Biostatistics and Bioinformatics (KEBB), Academic Medical Center of the University of Amsterdam, Amsterdam, The Netherlands; University of California San Francisco, United States of America

## Abstract

**Background:**

DC-SIGN expressed by dendritic cells captures HIV-1 resulting in *trans*-infection of CD4^+^ T-lymphocytes. However, BSSL (bile-salt stimulated lipase) binding to DC-SIGN interferes with HIV-1 capture. DC-SIGN binding properties of BSSL associate with the polymorphic repeated motif of *BSSL* exon 11. Furthermore, BSSL binds to HIV-1 co-receptor CXCR4. We hypothesized that BSSL modulates HIV-1 disease progression and emergence of CXCR4 using HIV-1 (X4) variants.

**Results:**

The relation between *BSSL* genotype and HIV-1 disease progression and emergence of X4 variants was studied using Kaplan Meier and multivariate Cox proportional hazard analysis in a cohort of HIV-1 infected men having sex with men (n = 334, with n = 130 seroconverters). We analyzed the association of *BSSL* genotype with set-point viral load and CD4 cell count, both pre-infection and post-infection at viral set-point. The number of repeats in BSSL exon 11 were highly variable ranging from 10 to 18 in seropositive individuals and from 5–17 in HRSN with 16 repeats being dominant (>80% carry at least one allele with 16 repeats). We defined 16 to 18 repeats as high (H) and less than 16 repeats as low (L) repeat numbers. Homozygosity for the high (H) repeat number *BSSL* genotype (HH) correlated with high CD4 cell numbers prior to infection (p = 0.007). In HIV-1 patients, delayed disease progression was linked to the HH *BSSL* genotype (RH = 0.462 CI = 0.282–0.757, p = 0.002) as was delayed emergence of X4 variants (RH = 0.525, 95% CI = 0.290–0.953, p = 0.034). The LH *BSSL* genotype, previously found to be associated with enhanced DC-SIGN binding of human milk, was identified to correlate with accelerated disease progression in our cohort of HIV-1 infected MSM (RH = 0.517, 95% CI = 0.328–0.818, p = 0.005).

**Conclusion:**

We identify *BSSL* as a marker for HIV-1 disease progression and emergence of X4 variants. Additionally, we identified a relation between *BSSL* genotype and CD4 cell counts prior to infection.

## Introduction

Millions have been infected worldwide with human immunodeficiency virus type-1 (HIV-1) but major differences exist between individuals in infection risk and rate of disease progression. These differences can be attributed in part to polymorphisms in host genes encoding HIV-1 co-receptors such as CCR5 and CCR2 and their ligands [1]–[5]. In addition, carrying human leukocyte antigen (HLA) variants B27 or B57 is associated with slower HIV-1 disease progression probably through HIV-1 specific CD8^+^ T-lymphocyte responses [6]. Knowledge of host genetic polymorphisms that affect HIV-1 pathogenesis has helped with the successful development of antiretroviral drugs and ways to modulate beneficial immune responses. However, due to therapy failure and incomplete virus suppression, there is a need for identification of novel drug targets for the treatment of HIV-1 infection.

Among cell types targeted by HIV-1 are dendritic cells (DCs), which play a central role in the protection against infections and the activation of anti-microbial immune responses [7]. DCs capture micro-organisms with the aim of degrading these pathogens and presenting their antigens to resting T-lymphocytes thereby activating adaptive immunity. Interaction between HIV-1 and DCs in all likelihood will occur at sites where mucosa is breached when individuals are exposed to HIV-1. DCs efficiently capture HIV-1 but captured HIV-1 partly escapes from degradation and fully infectious virus particles are subsequently presented to CD4^+^ T-lymphocytes, thereby efficiently infecting these target cells (*trans*-infection) [8].

DC-specific intercellular adhesion molecule-3 grabbing non integrin (DC-SIGN) is the major HIV-1 binding receptor expressed by immature DCs used for *trans*-infection. DC-SIGN forms tetramers that bind terminal fucoses and high mannose structures of “self” antigens and pathogen glycans and recognizes a wide range of micro-organisms [9]–[12]. HIV-1 binds to DC-SIGN and uses the pathogen receptor for *trans*-infection but, in addition, DC-SIGN binding of HIV-1 results in modulation of DC immune signaling [13]. DC-SIGN is also required for the formation of the infectious synapse between DC and T-lymphocyte during *trans*-infection of CD4^+^ T-lymphocytes with HIV-1 and DC-SIGN binding of neutralized virus can result in removal of neutralizing antibodies [14], [15]. Human DC-SIGN gene polymorphisms associate with the efficiency of HIV-1 transmission and the rate of disease progression and decreased DC-SIGN expression in Rhesus Macaques is associated with accelerated disease progression [16]–[18]. Whether DC-SIGN ultimately limits or accelerates human disease progression remains unclear, but obviously factors that competitively bind with HIV-1 to DC-SIGN will influence its function.

Among molecules that bind to DC-SIGN are mucin 6 in seminal plasma and bile-salt stimulated lipase (BSSL, BSDL, CEL) as well as mucin 1 identified in human milk [19]–[22]. BSSL is a dimeric glycoprotein that is abundantly expressed in milk but, in addition, is also expressed in blood [23], [24]. The presence of DC-SIGN blocking molecules in semen and breast milk may, at least in part, influence HIV-1 transmission. This could help explain the relatively low risk of HIV-1 infection during sex or breastfeeding [25]–[28]. We previously identified that breast milks derived from different mothers do not have equal DC-SIGN binding properties suggesting mother-dependent protection levels for HIV-1 transmission during breastfeeding [20]. In addition to DC-SIGN binding, BSSL from blood can bind to chemokine receptor CXCR4 [23], [29] suggesting a potential supplementary role for BSSL in HIV-1 pathogenesis.

Previous studies in our lab demonstrated that the DC-SIGN binding capacity of milk is associated with variation in the *BSSL* repeat domain [30]. Aim of the present investigation is to determine the relation between variation in the *BSSL* genotype (repeat domain) and HIV-1 infection and disease progression for men having sex with men (MSM) participating in the Amsterdam Cohort Studies on HIV infection and AIDS (ACS). This cohort contains a high number of seroconverters (n = 130) with a relatively long duration of follow-up in the pre-combination antiretroviral therapy (cART) era. Our research demonstrates that both slow HIV-1 disease progression and emergence of delayed CXCR4-using HIV-1 variants are correlated with homozygosity for the high (H) repeat number *BSSL* genotype (HH). The *BSSL* genotype that is dominant in patients with slow disease progression (HH) corresponds to the main genotype observed in high risk seronegative individuals. In addition, our study reveals that the *BSSL* HH genotype correlates with high CD4 cell numbers prior to infection. Our previous studies demonstrated that the LH *BSSL* genotype is associated with enhanced DC-SIGN binding of human milk [30]. In the present study on HIV-1 infected MSM we find that this LH genotype is associated with accelerated disease progression. Our research provides new insights in the role of BSSL in HIV-1 pathogenesis with the potential for BSSL as a new target for anti-HIV-1 drug development.

## Results

### Highly Exposed Seronegative Individuals and Slow Progressing Seropositives have Comparable *BSSL* Genotype Distributions

**Figure 1:**
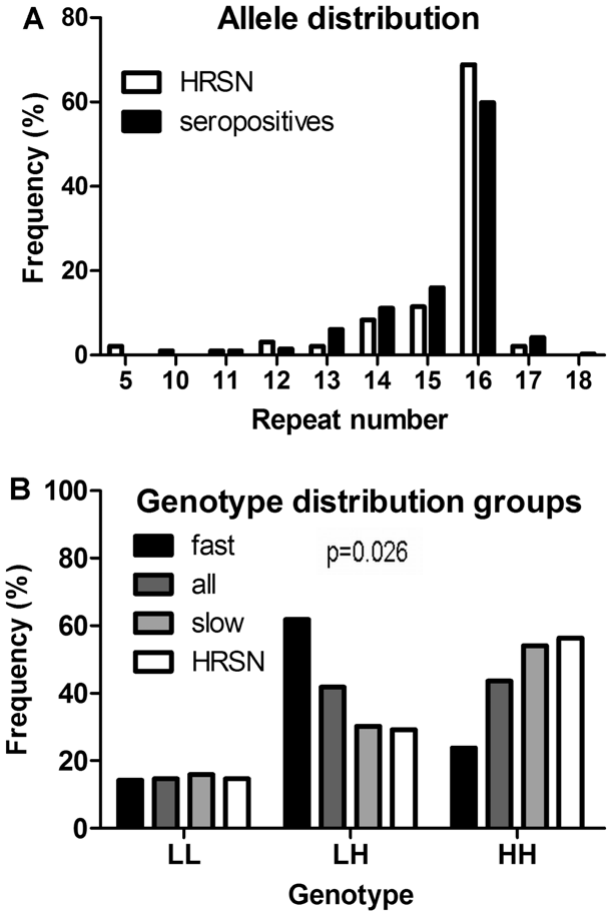
*BSSL* genotype distribution. (A) *BSSL* genotype distribution in high risk seronegative (HRSN, n = 48) individuals and HIV-1 positive individuals (n = 334). (B) Within the group of HIV-1 positives, individuals with fast disease progression (progression to AIDS or CD4 cell count <200 cells/µl within 3 years after seroconversion, n = 21) were compared to individuals with slow disease progression (time to AIDS or CD4 cell count <200 cells/µl is 10 years or longer after seroconversion, n = 63). We pre-defined 16–18 repeats as high (H) and less than 16 repeats as low (L) repeat numbers. *BSSL* genotype frequencies were compared for individuals with two low (LL), one low and one high (LH) or two high repeat numbers (HH). BSSL genotype distributions were different between individuals with slow disease progression when compared to individuals with fast disease progression (Pearson Chi-square p = 0.026). Additionally, HRSN were compared to the whole study population of seropositive individuals (all) for the LL, LH and HH genotypes (p = 0.190).

We genotyped the variable number of tandem repeat (VNTR) domain of the *BSSL* gene in a cohort of 334 HIV-1 seropositive men and 48 seronegative men with high risk behavior (high risk seronegatives, HRSN). We found that the number of repeats in this domain is highly variable ranging from 10 to 18 in seropositive individuals (Figure 1A). Furthermore we observed that 82% within this group carry at least one allele with 16 repeats, similar to the situation in previously genotyped HIV-1 negative mothers in a study on BSSL in human milk [30]. *BSSL* repeat numbers in HRSN range from 5–17 with 85% having at least one allele with 16 repeats (Figure 1A). As compared to seropositives, a trend was observed with HRSN having more frequently two alleles with 16 repeats (p = 0.059).

We hypothesized that specific *BSSL* genotypes might provide a certain level of protection against HIV-1 infection. To test this we categorized individuals in this study based on the number of repeats in *BSSL* exon 11. We pre-defined 16 to 18 repeats as high (H) and less than 16 repeats as low (L) repeat numbers as previously specified in our study of BSSL in human milk [30]. Individuals were categorized in 3 groups having either two low (LL), one low and one high (LH) or two high repeat number alleles (HH). HH is the major genotype whereas the LL genotype reaches relatively low numbers in both HRSN and seropositives. HRSN (n = 48) and whole study population of seropositive individuals (n = 334) were not statistically different for the HH genotype (Figure 1 B, p = 0.190).

Disease progression was defined as the time between HIV-1 infection and diagnosis of AIDS or when CD4 counts dropped below 200 cells/µl. Within the group of 334 seropositive individuals we compared the *BSSL* genotype for individuals progressing to AIDS within 3 years (fast progressors, n = 21) with individuals that remained AIDS free for 10 years or longer (slow progressors, n = 63) (Table 1). Figure 1B demonstrates that HRSN men and seropositive men with slow disease progression have similar *BSSL* genotype distributions.

**Table 1:** Characteristics of HIV-1 positive groups including fast progressors, slow progressors and whole group.

	Status	Age at SC^1^	Number infected	Ethnicity		Time to AIDS		CD4 at viral set-point		Viral load at set-point	
	Mean (yrs)	Range (yrs)		E^2^	non-E^2^	Median (yrs)	Range (yrs)	Mean (cells/ml)	SD^3^	Mean (Log RNA)	SD^3^
Fast progresssors	36.2	24.0–54.0	21	21	0	2.3	0.5–3.0	306	166	5	0.7
Slow progressors	32.1	20.9–48.6	63	61	2	12.7	≥10.0	631	235	3.7	0.7
All seropositives	34.9	19.6–55.6	334	315	19	6.0	0.5–≥13.0	554	263	4.2	0.8

1 seroconversion.

2 [Western] European descent.

3 standard deviation.

### *BSSL* Number of Repeats Correlates with HIV-1 Disease Progression

We compared the number of repeats in the VNTR domain of *BSSL* for fast progressors to that of slow progressors (Figure 1). The distribution of genotypes is significantly different between the fast and slow progressors (p = 0.026) with slow progressors having more frequently the HH genotype (Figure 1B, p = 0.016). The observed differences between fast and slow progressors did not change significantly after excluding the two non-Europeans from the slow progressor group (p = 0.21 and p = 0.12). Similar to the situation with slow progressors, HRSN more frequently carry the HH genotype when compared to fast progressors (p = 0.028).

**Figure 2:**
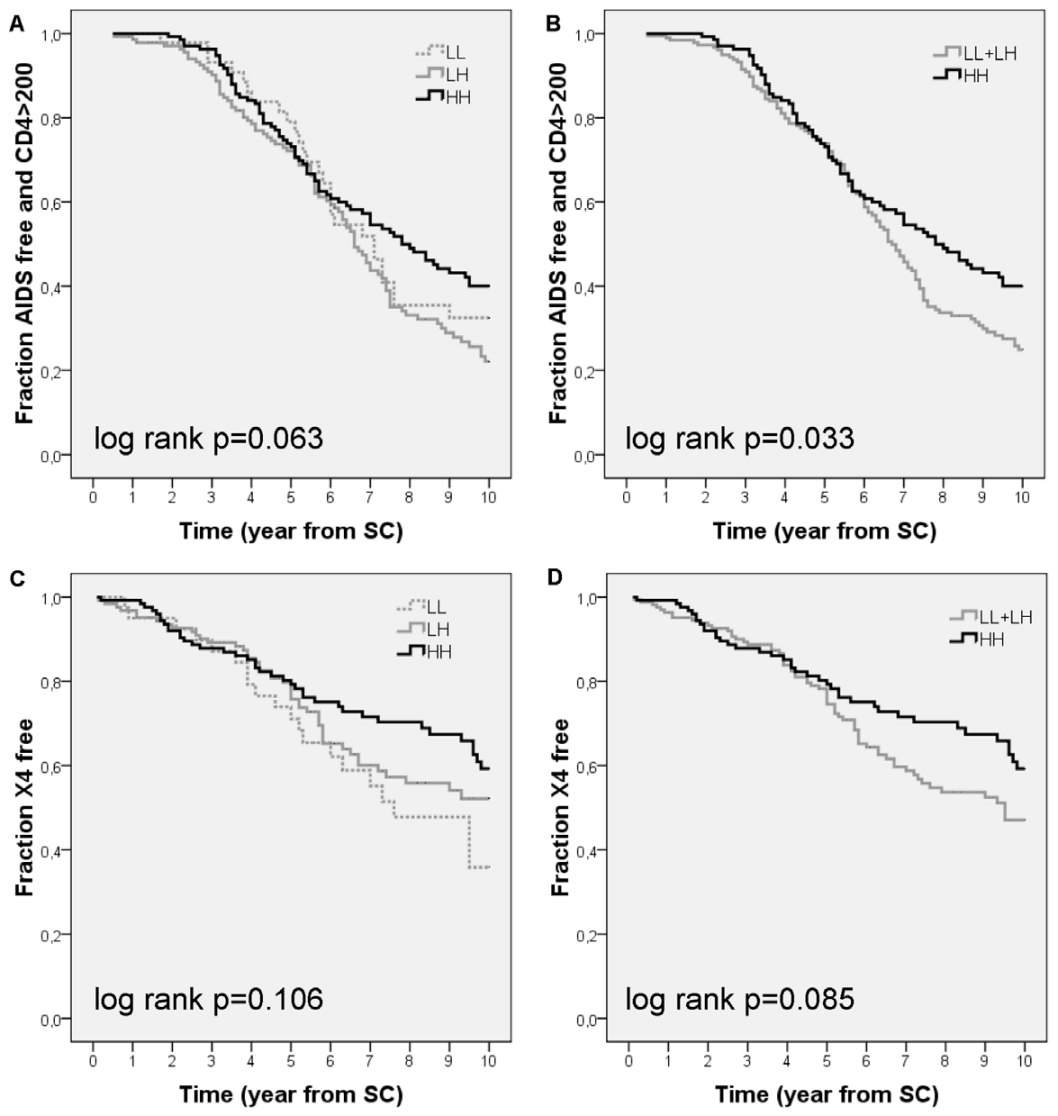
AIDS-free and CXCR4-free survival from HIV-1 seroconversion with and without *BSSL* HH genotype. Kaplan Meier estimation of patients with HH and non-HH (LL+LH) genotypes using (A+B) AIDS-free survival plotted for (A) all genotypes (LL n = 47, LH n = 139, HH n = 139, log rank p = 0.063), (B) HH versus LL+LH genotypes (HH n = 139, non-HH n = 186) (log rank p = 0.033) or (C+D) first detection of CXCR4-using HIV-1 variants as an endpoint for (C) all genotypes (LL n = 41, LH n = 126, HH n = 127, log rank p = 0.106) or (D) HH versus LL+LH genotypes (HH n = 127, non-HH n = 167, log rank p = 0.085).

Using Kaplan Meier methods we observed a trend towards differences in disease progression for patients with variable *BSSL* genotypes (Figure 2A, log rank p = 0.063). However, the effect of the different *BSSL* genotypes on disease progression appeared non-proportional and was observed from 6 years after seroconversion onwards. Cox proportional hazard analysis from 6 years post-seroconversion onwards demonstrates delayed disease progression in patients with the HH genotype (HH vs LH: RH = 0.436, 95% CI = 0.260–0.732, p = 0.002; HH vs LL: RH = 0.545, 95% CI = 0.271–1.095, p = 0.089; Table 2). Subsequent multivariate analysis from 6 years after seroconversion onwards demonstrated that the observed differences are independent of disease progression markers CCR5-Δ32, HLA-B57 and HIV-1 RNA and CD4 levels at viral setpoint (Table 2).

**Table 2:** Cox proportional hazard analysis to AIDS or CD4 cell counts below 200 cells/µl blood.

			Crude (6 years)				Adjusted (6 years)		
Comparison	Number^1^	Event^2^	RH^3^	CI^4^ (95%)	p	Number	RH	CI^4^ (95%)	p
BSSL HH vs LH	143	62	0.436	0.260–0.732	0.002	140	0.389	0.231–0.656	<0.001
BSSL HH vs LL	99	35	0.545	0.271–1.095	0.089	98	0.539	0.267–1.086	0.084
BSSL LH vs LL	96	51	0.801	0.419–1.529	0.501	94	0.722	0.375–1.389	0.329
CCR5-Δ32 vs WT^5^	168	74	0.457	0.251–0.832	0.010	166	0.451	0.247–0.826	0.010
HLA-B57	169	74	0.296	0.093–0.941	0.039	166	0.294	0.091–0.947	0.040
Viral RNA load (<10^4.5^ copies/ml)	167	73	0.571	0.338–0.966	0.037	166	0.642	0.376–1.097	0.105
CD4 cells (>500 cells/µl)	163	72	0.681	0.426–1.090	0.110	-	-	-	-

Cox proportional hazard analysis from 6 years after seroconversion to AIDS or CD4 cell counts below 200 cells per µl blood.

1 number of individuals included in the analysis.

2 number of individuals that reach the end point.

3 relative hazard.

4 95% confidence interval.

5 wild type CCR5 genotype.

Disease progression in patients with the LL genotype is not significantly different from that in patients with the LH (p = 0.501) or HH (p = 0.089) genotype, possibly due to the relatively low number of individuals carrying this genotype (Table 2). Since the LL genotype reaches relatively low frequencies we compared the major (HH) genotype with the combined LL with LH genotype (LL+LH) using Kaplan Meier methods. The results confirmed that the HH genotype associates with delayed disease progression (Figure 2B, log rank p = 0.033). No differences in disease progression were observed between groups until 6 years after seroconversion (RH = 0.967, 95% CI = 0.667–1.401). However, Cox proportional hazard analysis from 6 years post-seroconversion onwards confirmed the delayed disease progression observed in patients with the HH genotype (*BSSL* HH vs LL+LH, RH = 0.462 CI = 0.282–0.757, p = 0.002). Multivariate analysis demonstrated that the predictive value of the HH *BSSL* genotype for prolonged survival in this analysis was independent of the CCR5-Δ32 and HLA-B57 genotype and low viral load at viral set-point (RH = 0.425, 95% CI = 0.259–0.699, p = 0.001). Inclusion of ethnicity into the analysis did not alter the conclusions drawn (RH = 0.426, 95% CI = 0.258–0.701, p = 0.001).

**Figure 3:**
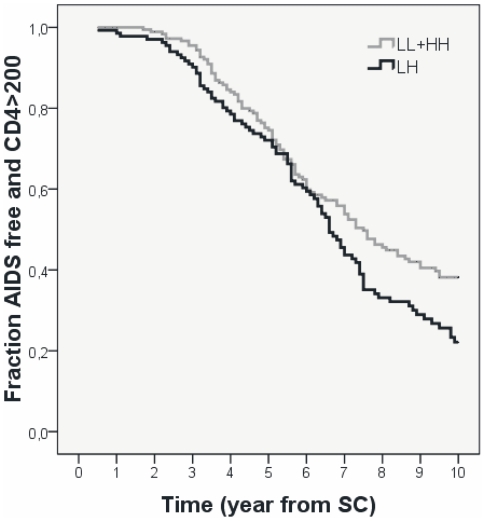
AIDS-free survival from HIV-1 seroconversion with and without the *BSSL* LH genotype. Kaplan Meier estimation of patients with LH and non-LH (LL+HH) genotypes using AIDS-free survival plotted LH versus LL+HH genotypes (log rank p = 0.025).

We previously observed enhanced DC-SIGN binding properties of human milk derived from mothers carrying the LH *BSSL* genotype [30]. In the present study on HIV-1 infected MSM we observed a significant difference in disease progression between individuals with the LH and HH genotypes (Table 2). To test the predictive value of the LH genotype for disease progression, we combined the patients having the LL and HH genotypes and compared disease progression for these patients with patients having the LH genotype. Using Kaplan Meier methods we observed that the LH genotype is associated with accelerated disease progression (Figure 3, log rank p = 0.025). Cox proportional hazard analysis from 6 years post-seroconversion onwards confirmed the association between LH genotype and accelerated disease progression (RH = 0.517, 95% CI = 0.328–0.818, p = 0.005).

### Emergence of CXCR4-Using Variants is Associated with the Number of Repeats in *BSSL*

DC-SIGN differentially enhances *in-trans* infection with CCR5 (R5) and CXCR4 using (X4) HIV-1 and BSSL protein interacts with CXCR4 [23], [31]. We hypothesized that size variation in the *BSSL* exon 11 repeat domain could influence the time to X4 emergence during disease progression. Using Kaplan-Meier methods we observed no significant effect of the three *BSSL* genotypes on X4 emergence (Figure 2C, log rank p = 0.106) although patients with the HH genotype trended towards delay in X4 emergence when compared to patients with the combined LL+LH genotypes (Figure 2D, log rank p = 0.085). However Figures 2C and D suggested effects of the *BSSL* genotypes on X4 emergence appear non-proportional and were observed from 5 years post-seroconversion onwards. Cox proportional hazard analysis from 5 years onwards demonstrated delayed emergence of X4 variants in patients with the HH genotype (HH vs LL: RH = 0.418, 95% CI = 0.194–0.901, p = 0.026; HH vs LH: RH = 0.577, 95% CI = 0.304–1.094, p = 0.092).

When we compared the major (HH) genotype with the combined LL with LH genotype (LL+LH) using Cox proportional hazard analysis we confirmed the protective effect for the HH genotype (RH = 0.525, 95% CI = 0.290–0.953, p = 0.034). When comparing the LH genotype with the combined LL with HH genotype (LL+HH) using Kaplan-Meier methods we observed no effect on X4 emergence from the LH genotype (log rank p = 0.626). Multivariate analysis demonstrated that the predictive value of the HH *BSSL* genotype for X4 emergence in this analysis was independent of ethnicity (RH = 0.541, 95% CI = 0.297–0.985, p = 0.044). For the individuals remaining AIDS free and those remaining X4 free we analyzed the relationship between clinical progression to AIDS and X4 emergence within these groups. We observed that 86% of the individuals remaining AIDS free also did not develop X4 variants with no significant difference between the HH and non-HH-genotypes (p = 0.47).

### *BSSL* Genotype is Associated with CD4 Cell Numbers in Uninfected Individuals

**Figure 4:**
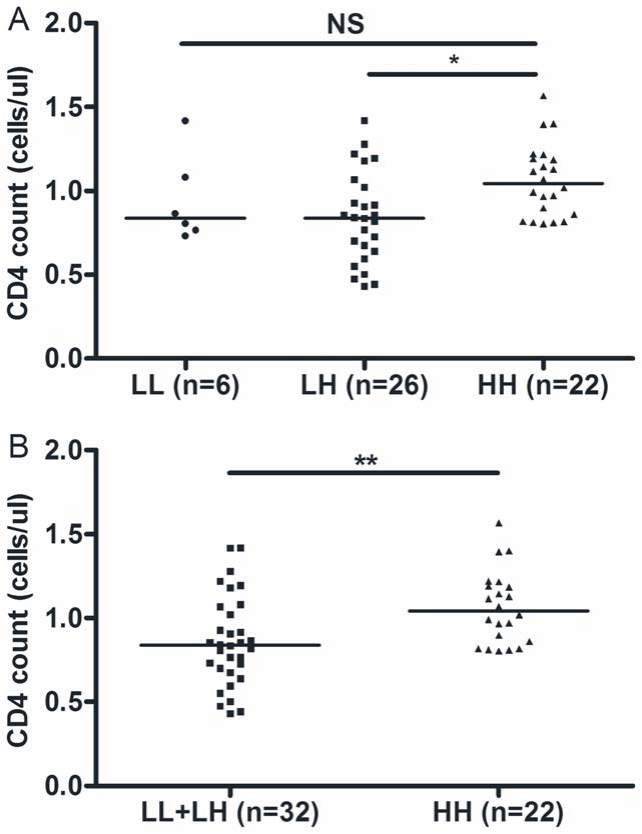
*BSSL* HH genotype is associated with high CD4 cell numbers pre-seroconversion. The relation between *BSSL* genotypes and CD4 cell count before seroconversion for individuals with the (A) LL, LH and HH genotypes (p = 0.022) and (B) HH versus non-HH (LL+LH) genotypes (p = 0.007). Median values are indicated with a horizontal line. NS: not significant, *: p<0.05, **, p<0.01.

We studied the relation between *BSSL* genotype and the HIV-1 viral load and CD4 cell count at viral setpoint, but did not find significant differences between patients with different *BSSL* genotypes (data not shown). Furthermore, we observed no significant differences in CD4 decline from viral setpoint onwards for the different genotypes (data not shown). However, within the group of individuals for whom CD4 cell counts prior to infection were known, we found that CD4 cell numbers prior to infection were elevated within the group of individuals carrying the HH genotype (p = 0.022, Figure 4A). Comparing the HH genotype with the combined LL with LH genotypes (LL+LH) confirmed the elevated CD4 cell numbers prior to infection for individuals with the HH genotype (p = 0.007, Figure 4B).

## Discussion

In this study we demonstrate that the rate of HIV-1 disease progression is associated with *BSSL* polymorphisms in a cohort of MSM. *BSSL* genes have either a high (H = 16 to 18) or a low (L<16) number of repeats in their variable number of tandem repeat (VNTR) domain. Individuals can be categorized into having two low (LL), one low and one high (LH) or two high (HH) repeat number *BSSL* alleles. Our study reveals that uninfected individuals with the HH genotype have elevated CD4 cell numbers compared to individuals carrying the other genotypes. Once infected with HIV-1, MSM with the HH genotype progress to disease more slowly and show delayed development of CXCR4-using HIV-1 variants.

Since differences in DC-SIGN binding properties of human breast milk link to differently sized BSSL forms [20], [30], we hypothesized that BSSL expressed in blood behaves in a similar fashion. We demonstrate here that variation in the number of repeats encoded by the *BSSL* gene is linked to disease progression in HIV-1 infected MSM. This could be the result of differences in DC-SIGN binding by differently sized BSSL forms. The LH genotype is associated with increased DC-SIGN binding capacity of human milk [30] whereas in the present study we find this LH genotype associated with accelerated disease progression. This could imply that increased DC-SIGN blocking and thus decreased DC-SIGN availability may accelerate disease progression similar to the situation with low DC-SIGN expression in Rhesus macaques. But care should be taken when translating the DC-SIGN binding properties of BSSL from milk into those of BSSL in blood since post-translational modifications such as glycosylation, essential for DC-SIGN binding, may differ significantly.

In line with the effect of the *BSSL* genotype on progression to AIDS, we observed a delay in the emergence of CXCR4-using HIV-1 variants is associated with the HH genotype. Both disease progression and emergence of CXCR4-using variants are delayed in patients with the HH genotype, although this might in part reflect the relation between the emergence of CXCR4-using variants and disease progression [32].

Much of the damage related to HIV-1 infection, including rapid depletion of CD4 cell numbers in the gut, occurs early in infection whereas clinical manifestation are only detected years later. Additionally, factors such as infecting viral strain phenotype, CCR5-Δ32 and HLA genotypes can influence virulence during the early stages of infection. This may explain why at viral setpoint no significant differences were observed in CD4 cell numbers and viral loads between carriers of different BSSL genotypes while differences in CD4 cell numbers prior to seroconversion existed and re-emergenced 6 years after seroconversion. Furthermore, if BSSL indeed interacts with CXCR4 resulting in differential emergence of CXCR4-using variants it is expected that the effects appear later in disease.

DC-SIGN has been suggested to differentially enhance *trans*-infection with R5 and X4 virus and the formation of infectious synapses between DCs and T-lymphocytes and subsequent T-cell stimulation [14], [31]. Furthermore, CXCR4 not only acts as co-receptor for CXCR4-using HIV-1 infection but is also involved in the regulation of T-cell migration, proliferation and differentiation [5], [33], [34]. BSSL interacts with both DC-SIGN and CXCR4 although not via the same structural domain [20], [23]. Differences in *BSSL* genotypes in all likelihood translate into BSSL proteins with different DC-SIGN or CXCR4 binding properties and related modulation of DC-SIGN and CXCR4 roles in disease progression and CXCR4-using variant emergence. However, more research is needed to identify whether disease progression and emergence of CXCR4-using variants are similarly influenced by these interactions. In addition, differences in the interaction between BSSL and CXCR4 in individuals with different *BSSL* genotypes might influence CD4 cell homeostasis.

The interaction between BSSL and CXCR4 was proposed to be mediated through the V3 like domain of BSSL, which is not located in the VNTR region [29]. This suggests that the potential influence of BSSL size variation on the interaction between BSSL and CXCR4 could be indirect, for example by influencing the presentation of the V3 like domain to CXCR4. In addition, there may also be structural constraints that influence plasma concentrations of the different BSSL forms. In vitro studies with differently sized BSSL proteins should provide a better insight in the role of the VNTR in the interaction between BSSL and CXCR4.

The *BSSL* genotype distribution in HRSN was similar to that of the slow progressors with a relative abundance of the HH genotype. We suggest that the HH genotype provides partial protection against infection possibly related to heightened peripheral blood CD4 cell numbers in carriers of this genotype. However, more research with higher numbers of HRSN included is needed to further clarify the potential protective effect of BSSL in protection against HIV-1 infection.

Taken together, we have identified *BSSL* as a marker for HIV-1 progression to AIDS and the emergence of CXCR4-using viruses in HIV-1 infected MSM. Furthermore, we revealed an association between *BSSL* genotypes and CD4 cell count in blood of uninfected individuals. Further investigation should be aimed at characterizing the effects of differently sized BSSL proteins on the interaction between DC-SIGN and serum derived BSSL. In addition, the binding between BSSL and CXCR4 should be further characterized including the effect of such binding on infection with CXCR4-using variants, CXCR4 signaling and CD4^+^ T-lymphocyte proliferation and migration. These studies may ultimately increase our understanding of the role of BSSL in HIV-1 pathogenesis.

## Materials and Methods

### Study participants and ethics statement

We studied HIV-1 infected men having sex with men (MSM) as well as seronegative MSM with high risk behavior (high risk seronegatives, HRSN). The MSM were enrolled in the Amsterdam Cohort Studies on HIV infection and AIDS (ACS) between October 1984 and March 1986 as previously described [35]. In short, our study included 50 HRSN and HIV-1 seropositive MSM and DNA was available for 335 HIV-1 infected and 48 HRSN. HRSN had a HIV-seronegative follow-up despite unprotected receptive anogenital sex with at least 2 different partners and/or a reported episode of syphilis [36]. Among HIV-1 infected, 130 MSM seroconverted during follow-up and 205 tested positive for HIV-1 antibodies at entry in the cohort of whom the date of seroconversion can be reliably estimated. PCR failed with one of the HIV-1 positive individuals and this sample was therefore excluded from further analysis. The ACS has been conducted in accordance with the ethical principles set out in the declaration of Helsinki and written informed consent is obtained prior to data collection. The study was approved by the Amsterdam Medical Center institutional medical ethics committee.

### Variables and definition of Disease Progression and Emergence of CXCR4 Variants

Progression to AIDS and emergence of CXCR4 using variants were studied. AIDS was defined as clinical AIDS or a CD4 cell number below 200 cells/µl according to the 1993 CDC definition [37]. CXCR4 using HIV-1 variants were detected by co-culture of patient PBMC and MT2 cells as previously described [38].

### Viral Load and CD4 Cell Count

Methods for viral load and CD4 cell counts determination from individuals in the study population were described previously [35]. A value for CD4 cell count pre seroconversion was determined only when three or more CD4 measurement time points were available. CD4 cell count pre seroconversion was defined by the mean value of all the available measurements when HIV-1 negative.

Viral load and CD4 cell count at viral set-point (2 years after seroconversion) were included as disease progression markers. Multivariate analysis that included viral load and CD4 cell count at viral setpoint were performed from 2 years after seroconversion onwards. HIV-1 set-point viral load below 10^4.5^ copies per ml plasma was pre-defined as low viral load and CD4 levels at viral set-point above 500 cells per µl blood were pre-defined as high CD4 cell numbers.

### Genotyping PCR

Primers specific for *BSSL* were designed (forward primer: ACC AAC TTC CTG CGC TAC TGG ACC CTC, reverse primer: TGA TAC CAA GGC TCA TGG GAC GCT AAA AC) containing a FAM label for detection. After initial denaturation for 4′ at 94°C the following PCR program was run for 35 cycles: 30″ 94°C, 3′ 60°C, 1′ 72°C followed by an extended elongation for 7′ at 72°C. The PCR product was mixed with the Genescan™ – 1200 LIZ® Standard (Applied Biosystems; catalog#: 4379950) and the ABI 3100 capillary sequencer (Applied Biosystems) in Genescan mode was used for PCR product size determination. Data was analyzed using Genemapper software (Applied Biosystems).

### Statistical Analysis

Kaplan Meier methods and multivariate Cox proportional hazard analysis were used to study the relation between *BSSL* genotype and progression to AIDS and the emergence of CXCR4-using variants. Follow-up was calculated from the estimated date of seroconversion until the earliest date of AIDS/emergence of CXCR4 using variants (events), loss to follow-up, death or 1 January 1996 (censors). Patients dying of non AIDS-related causes were censored at the time of death. Since the introduction of cART in the Netherlands in 1996 will likely influence disease course and emergence of CXCR4-using variants in infected individuals the censor data were set at 1-1-1996 thereby excluding post-cART era data points. Pearson Chi-square exact test was used to study the relation of *BSSL* genotype distributions between HRSN and seropositives as well as between fast and slow progressors. First, overall differences in genotype distributions were studied. When significant effects were found a posthoc test was used to determine the source of the observed differences. The effect of the genotype on CD4 cell count pre seroconversion, CD4 set-point and viral load set-point was analyzed using a non-parametric ANOVA (rank transformed values). All Kaplan Meier, Cox proportional hazard analyses and Pearson Chi-square exact tests were carried out using SPSS (release 15, SPSS Inc.) and all ANOVA analyses using Graphpad Prism software version 5. P-value<0.05 was considered statistically significant.
